# Detection and characterization of a coxsackievirus B2 strain associated with acute meningoencephalitis, Brazil, 2018

**DOI:** 10.1590/0037-8682-0499-2019

**Published:** 2020-11-13

**Authors:** Ivanildo Pedro de Sousa, Raiana Scerni Machado, Fernanda Marcicano Burlandy, Edson Elias da Silva

**Affiliations:** 1 Fundação Oswaldo Cruz, Instituto Oswaldo Cruz, Laboratório de Enterovírus, Rio de Janeiro, RJ, Brasil.

**Keywords:** Meningoencephalitis, Enterovirus, Coxsackievirus B2

## Abstract

Although different etiological agents can cause acute meningoencephalitis, this syndrome is usually associated with viruses. Among these, enteroviruses play a significant role. Here, we describe a fatal case of meningoencephalitis in a previously healthy teenager. Real-time RT-PCR and cell culture assays were performed with serum and cerebrospinal fluid (CSF) from a clinically diagnosed meningoencephalitis case that occurred in Rio de Janeiro State, Brazil. Coxsackievirus B2 (CVB2) was identified. Phylogenetic analysis revealed that the identified CVB2 was genetically related to strains known to cause neurological diseases. This case highlights the importance of continuous laboratory surveillance of central nervous system infections.

## INTRODUCTION

The human *Enterovirus* genus, *Picornaviridae* family, comprises seven species (EV A-D and HRV A-C) which show great genetic variability, and can cause a broad spectrum of acute and chronic infections ranging from asymptomatic or mild illnesses to acute flaccid paralysis, meningitis, and meningoencephalitis^1^. Although meningoencephalitis can be caused by a large number of infectious agents, such as bacteria and fungi, in the vast majority of cases, viruses are the causative pathogens^2^. Among these, enteroviruses are indicated to be the main causative agents of central nervous system infections in neonates and immunocompromised^3^ ^,^ ^4^. 

Meningoencephalitis is a severe inflammatory process of the brain parenchyma and the meninges, causing fever, severe headache, seizures, and in some cases, neurological damage. Enteroviral meningoencephalitis is relatively well documented around the world, although most studies have focused on ‘epidemic’ EV-types rather than ‘sporadic’ ones, such as CVB2. To date, only one case of CVB2 causing meningoencephalitis has been reported in Brazil, based on serological evidence^5^. Thus, information regarding the molecular epidemiology of this agent is limited. Here, we report the detection and molecular characterization of a fatal case of CVB2 associated with meningoencephalitis in a previously healthy teenager, and the genetic relationship of the isolated CBV2 with other isolates. 

## CASE REPORT

**FIGURE 1:**
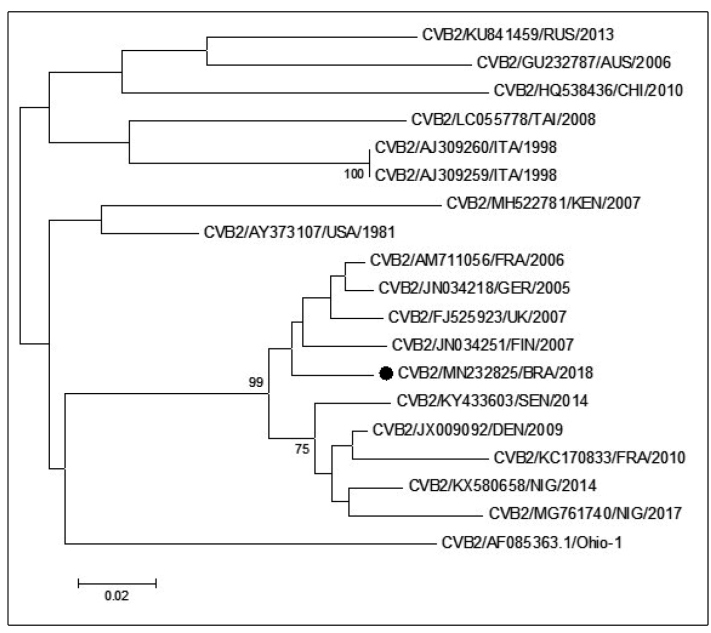
Phylogenetic analysis of the viral protein 1(VP1) gene fragment (240 nt.) of the CVB2 isolate (indicated by black circle) from a fatal case of meningoencephalitis in Brazil in 2018. The tree was generated from a nucleotide sequence alignment using the neighbor-joining algorithm implemented in MEGA 6.0 software using a Kimura two-parameter model and 1000 bootstrap pseudo replicates. Only bootstrap values >70% are shown at the node. The sequence in this study was submitted to GenBank under accession number MN232825. **RUS:** Russia; **AUS:** Australia; **CHI:** China; **TAI:** Taiwan; **ITA:** Italy; **KEN:** Kenya; **USA:** United States of America; **FRA:** France; **GER:** Germany; **UK:** United Kingdom; **FIN:** Finland; **BRA:** Brazil; **SEN:** Senegal; **DEN:** Denmark; **NIG:** Nigeria

In 2018, the Enterovirus laboratory received a group of specimens (CSF and serum) from a 16 year old patient, who presented at a health center in the Rio de Janeiro State with symptoms of meningoencephalitis, which was confirmed by imaging tests. The patient was undergoing treatment for acute sinusitis at the time of his admission to the health center. Specimens were submitted for virus isolation using two types of enterovirus-sensitive cell culture (RD (human rhabdomyosarcoma) and Hep2C (human cervix carcinoma)) as previously described^6^. Both cell cultures failed to reveal a cytopathic effect. Viral RNA was extracted directly from the specimens (Viral Nucleic Acid Extraction Kit, QIAmp-Qiagen) and used for detection by amplification methods. A broadly reactive real-time PCR assay for detection of enteroviral genomic RNA was performed, and the serum was enterovirus-positive. The specimen was then subjected to semi-nested PCR amplification as described previously^7^, and the EV-positive amplicon was cycle-sequenced using a Big Dye Terminator v3.1 Cycle Sequencing Kit (Applied Biosystems). The sequences obtained, which corresponded to an ~340bp fragment of the VP1 gene, were compared with those available in GenBank. Coxsackievirus B2 was identified in the patient’s serum. Phylogenetic analysis using the VP1 gene fragment sequence confirmed the close relationship (~95%) between the CVB2 identified in this study and European isolates associated with neurological diseases between 2000 and 2007 (Figure 1). The clinical specimens were also tested by PCR for HSV1/2, which were not detected. Serological tests for HSV, VZV, HCMV and toxoplasmosis were also negative. RT-PCR for arboviruses also revealed negative results (data not shown).

## DISCUSSION

Recent reports have described an increasing number of neurological syndromes associated with CVB2, such as acute flaccid paralysis, meningitis, encephalitis, meningoencephalitis, and other neurological manifestations^8^ ^,^ ^9^. Coxsackievirus may cause upper and lower respiratory tract disease^8^, but does not commonly result in central nervous system secondary infection, except among immunocompromised individuals^4^. This patient’s medical history did not reveal any significant previous disease. 

Here, we have shown that CVB2 was associated with a fatal case of meningoencephalitis in a 16 year old teenager. Between 2013 and2017, CVB2 has only been found once in Brazil as the cause of a central nervous system infection^10^. Although CVB2 can be associated with non-neurological diseases^8^, phylogenetic analysis of the CVB2 described in this study revealed a close relationship with CVB2 strains previously associated with neurological diseases in Europe (Figure 1).

Overall, enteroviral meningoencephalitis is considered to be a self-limiting infection, even though certain EV-types may lead to more complex clinical manifestations, such as those caused by EV-A71 and EV-B^9^ ^,^ ^11^. In this study, we show the importance of an established surveillance system to detect and report the emergence of specific enterovirus types with the potential to cause severe diseases. Continuous surveillance of enterovirus infections is essential to identify EV-types circulating in different regions, and to implement public health strategies for prevention. Furthermore, due to the high frequency of enterovirus intra and intergroup genetic recombination, it is important that the surveillance system remain active.

### Ethical Approval

The Enterovirus laboratory at Instituto Oswaldo Cruz - FIOCRUZ is an official Brazilian Ministry of Health Reference Laboratory. During the course of the patient’s clinical care, the patient voluntarily accessed the local public health system’s ambulatory care center, where clinical samples were collected. This activity was considered a public health response, and thus did not require review by the review board. 
